# A model postulating a pivotal role of the levator-depressor neuro-muscular systems in locomotion of the stick insect

**DOI:** 10.1186/1471-2202-16-S1-P49

**Published:** 2015-12-18

**Authors:** Tibor I Toth, Silvia Daun-Gruhn

**Affiliations:** 1 Heisenberg Research Group of Computational Biology, Institute of Zoology, University of Cologne, 50674 Cologne, Germany

## 

Locomotion of stick insects has been intensively studied for many years. These studies made use of both behavioural [1,2] and electrophysiological e.g. [3,4] methods. In addition, models have been constructed to mimic the locomotion (normal walking) of these insects e.g. [5]. All these studies have substantially advanced our knowledge on insect locomotion. Nevertheless, there still exist a large number of unresolved problems in this field. One of them is about the possibly hierarchical organization of the coordinated movements of the leg-joints during normal walking. The existing experimental data relating to this problem have not proved decisive. A modelling approach would therefore seem particularly apt to contribute to the resolution of this problem. The advantage of this method is that a wide range of scenarios and hypothetical experimental arrangements can be emulated by a model that bears the main hallmarks of its biological counterpart.

We followed exactly this route when investigating the organization of locomotion. We thus extended an earlier model of ours [6] that emulates the coordinated muscle activities in a single (middle) leg of the stick insect to include all three ipsilateral legs. The model of each leg consisted of the three main neuro-muscular systems that correspond to the three main antagonistic muscle pairs: m. protractor and retractor coxae, m. levator and depressor trochanteris and m. extensor and flexor tibiae. The muscles were activated by corresponding motoneurones which, in turn, were controlled by the activity of non-spiking pre-motor interneurones and the activity of central pattern generators (CPGs). Our basic assumption was that the levator-depressor systems of each leg play a pivotal role in the organization of the coordinated movement of the three ipsilateral legs. According to our reasoning, the most reliable signal that affects normal stepping during locomotion is ground contact (touch-down) and lift-off. Both of them are, in essence, brought about by vertical movement of the femur of the leg, that is by proper activation of the levator-depressor neuro-muscular system.

We tested this assumption accordingly in simulations with the model. In doing so, we succeeded in mimicking both the tetrapod and the tripod coordination patterns, which stick insects exhibit during normal walking. Moreover, we found that the coordination process in tripod only requires position signals of the legs whereas in tetrapod angular velocity signals, too, are necessary for the coordination. We also succeeded in modelling the transition between these coordination patterns and found that the switch is, in some sense, easier from tripod to tetrapod than in the opposite direction. The model thus supports our assumption on the pivotal role of the levator-depressor neuro-muscular system in normal walking.
